# Highly Tunable Emission by Halide Engineering in Lead-Free Perovskite-Derivative Nanocrystals: The Cs_2_SnX_6_ (X = Cl, Br, Br/I, I) System

**DOI:** 10.3389/fchem.2020.00035

**Published:** 2020-01-31

**Authors:** Alessandro Veronese, Maddalena Patrini, Daniele Bajoni, Carlo Ciarrocchi, Paolo Quadrelli, Lorenzo Malavasi

**Affiliations:** ^1^Department of Physics and CNISM, University of Pavia, Pavia, Italy; ^2^Department of Electrical, Computer and Biomedical Engineering, University of Pavia, Pavia, Italy; ^3^Department of Chemistry and INSTM, University of Pavia, Pavia, Italy

**Keywords:** spectroscopy, lead-free, emission, perovskite, nanocrystal, nanocrystal (NC)

## Abstract

Nanocrystals of Cs_2_SnX_6_ (X = Cl, Br, Br_0.5_I_0.5_, and I) have been prepared by a simple, optimized, hot-injection method, reporting for the first time the synthesis of Cs_2_SnCl_6_, Cs_2_SnBr_6_, and mixed Cs_2_Sn(I_0.5_Br_0.5_)_6_ nanocrystalline samples. They all show a cubic crystal structure with a linear scaling of lattice parameter by changing the halide size. The prepared nanocrystals have spherical shape with average size from 3 to 6 nm depending on the nature of the halide and span an emission range from 444 nm (Cs_2_SnCl_6_) to 790 nm (Cs_2_SnI_6_) with a further modulation provided by mixed Br/I systems.

**Graphical Abstract:**
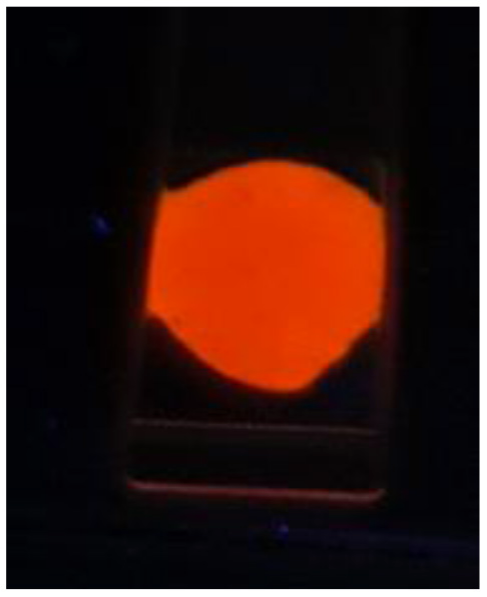
The series of Cs_2_SnX_6_ (X = Cl, Br, Br_0.5_I_0.5_, and I) nanocrystals with average size around 3 nm is reported with the first synthesis of Br, and mixed Br/I compounds. A progressive tuning of structural and optical properties is observed along with the anion composition. The nanocrystals are stable for several days under ambient conditions.

## Introduction

The extremely facile synthesis and the impressive optical properties of metal halide perovskites (MHPs) nanocrystals (NCs) make them an ideal class of materials for several optoelectronic devices, with the possibility of replacing, in the future, other conventional semiconductors in everyday life applications (Amgar et al., 2016; Huang et al., 2016, 2017; Kovalenko et al., 2017; Swarnkar et al., 2017). The major issue of current metal halide perovskites, such as CsPbX_3_ (X = Cl, Br, I), is the presence of lead, a toxic metal that raises a great concern in view of applications. While trying to overcome this issue, researchers are exploring new compounds that substitute lead with more environmental-friendly elements, e.g., tin, germanium, bismuth, palladium, or antimony (Jellicoe et al., 2016; Liu et al., 2017; Pal et al., 2017; Wang et al., 2017; Yang et al., 2017; Zhang et al., 2017; Bekenstein et al., 2018; Creutz et al., 2018; Leng et al., 2018; Sun et al., 2018; Wu et al., 2018; Zhou et al., 2018a,b). These alternative perovskites at the moment still present poorer optical properties as compared to lead-based ones. Nevertheless, they are of primary interest for the development of this research topic and they deserve more and deeper investigations, both theoretical and experimental, to improve their performances.

Tin halide perovskites represent a promising alternative to lead halide perovskites, especially for photovoltaic applications. Due to the higher electronegativity of tin, these compounds have indeed a narrower band-gap in comparison to their lead-containing counterparts, and so they are potentially better light harvesters (Mancini et al., 2015; Jellicoe et al., 2016; Ferrara et al., 2017; Pisanu et al., 2018). Tin-based perovskites, however, suffer from a severe chemical instability that heavily hampers the investigation of their physical properties. Under ambient conditions, Sn^2+^ tends to easily oxidize into the more stable Sn^4+^; the problem of oxidation is even more pronounced in the case of NCs because of the higher surface–volume ratio. This undesired transition is followed by the creation of trap states that irreversibly deteriorate the light emission properties of the crystals, thus suggesting a defect-intolerant nature of tin-based perovskites. Moreover, spin–orbit coupling of Sn 5*p* states is weaker compared to that of Pb 6*p* states, therefore their conduction band (CB) is less stabilized and the probability of formation of trap states is increased (Hermann et al., 2010; Umari et al., 2015).

A possible strategy to overcome this problem is to replace Sn^2+^ with Sn^4+^ in order to avoid its oxidation. This substitution modifies the standard perovskite crystal structure and leads to the formation of a so-called vacancy-ordered double perovskite. In this perovskite derivative, the B-sites are alternatively occupied by a tetravalent ion and vacancies, and the BX_6_ octahedra result thereby to be isolated (Cai et al., 2017; Dalpian et al., 2017; Ju et al., 2018). At present, studies on Sn^4+^-based double perovskites in the form of colloidal nanocrystals are still very limited and only the synthesis of Cs_2_SnI_6_ and, very recently, of Mn-doped Cs_2_SnCl_6_ NCs was reported by few groups employing different synthetic procedures (Wang et al., 2016; Dolzhnikov et al., 2017; Ghosh et al., 2018; Jing et al., 2019; Lin et al., 2019; Xu et al., 2019). Wang et al. (2016) demonstrated an enhancement in the chemical stability of Cs_2_SnI_6_ NCs compared to those based on Sn^2+^. The synthesis and purification of the crystals were performed under ambient conditions and, after 1 week of storage out of glove-box, the samples did not show degradation of the optical properties.

The interest on these perovskite Sn^4+^ containing materials is then relevant for basic and applied research and it would be of great importance to extend their optical properties through stoichiometry manipulation such as anion substitution, as already performed in other NCs systems (Jellicoe et al., 2016). To the best of our knowledge, only Cs_2_SnI_6_ NCs and Cs_2_SnCl_6_ have been described in the current literature in the past, while in the present paper we are reporting on the synthesis of the whole set of nanocrystalline Cs_2_SnX_6_ materials with X = Cl, Br, I. and mixed I/Br achieved by means of an optimization of the currently reported synthesis methods for Cs_2_SnI_6_ (Wang et al., 2016; Dolzhnikov et al., 2017; Ghosh et al., 2018; Xu et al., 2019). A clear and monotonic modulation of structural and optical properties with anion composition has been observed in the prepared Cs_2_SnX_6_ NCs, similarly to 3D-perovskite analogs, with a significant variation of the band gap from 1.58 eV (Cs_2_SnI_6_) to 3.86 eV (Cs_2_SnCl_6_) and emission properties in line or even better with respect to available reports on Cs_2_SnI_6_ (Wang et al., 2016; Dolzhnikov et al., 2017; Ghosh et al., 2018; Xu et al., 2019).

## Results and Discussion

**Figure 1:**
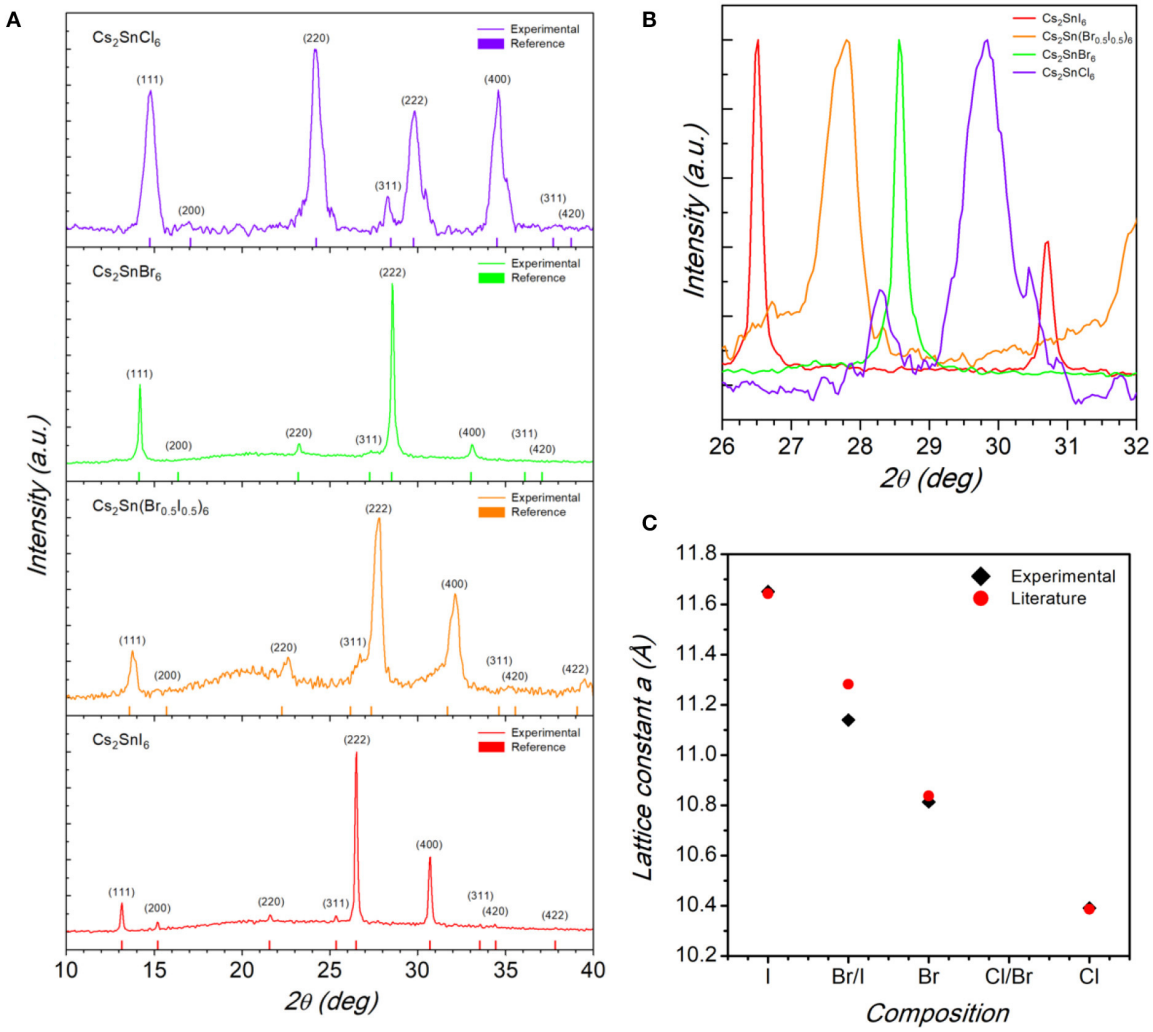
**(A)** Indexed X-ray diffraction patterns of the Cs_2_SnX_6_ (*X* = Cl, Br, Br_0.5_I_0.5_, and I) samples. Vertical bars in each pattern refer to the calculated cubic structure. **(B)** Pattern zoom in the region around the (222) reflection for the Cs_2_SnX_6_ samples highlighting the peak shift from higher to lower angle by replacing Cl for I. **(C)** Trend of the cubic lattice parameter as a function of chemical composition for the Cs_2_SnX_6_ samples.

Nanocrystalline Cs_2_SnX_6_ (X = Cl, Br, Br_0.5_I_0.5_, and I) samples have been synthesized according to the methodology reported in the Experimental Section through an optimized route with respect to the available ones (Jellicoe et al., 2016; Wang et al., 2016; Dolzhnikov et al., 2017; Ghosh et al., 2018; Xu et al., 2019). The laboratory powder X-ray diffraction (XRD) patterns of the Cs_2_SnX_6_ (X = Cl, Br, Br_0.5_I_0.5_, and I) samples are reported in Figure 1A.

From the patterns reported in Figure 1A it is possible to note that all the sample are single-phase and can be indexed according to the cubic *Fm-*3*m* space group, as previously reported for Cs_2_SnI_6_ and Cs_2_SnCl_6_ (Wang et al., 2016; Dolzhnikov et al., 2017; Jing et al., 2019; Lin et al., 2019; Xu et al., 2019). By a simple visual inspection of the main reflection in Figure 1B, i.e., the (222), a shift to higher 2θ values passing from Cs_2_SnI_6_ (bottom panel in Figure 1A) to Cs_2_SnCl_6_ (top panel) is clear, indicating a reduction of the cubic cell size by replacing I with Cl. The trend of cubic lattice parameter determined for the four samples is reported in Figure 1C, showing a significant change of the unit cell from 11.642(2) Å for Cs_2_SnI_6_ to 10.391(2) Å for Cs_2_SnCl_6_ with a reasonable linear scaling as a function of composition.

**Figure 2:**
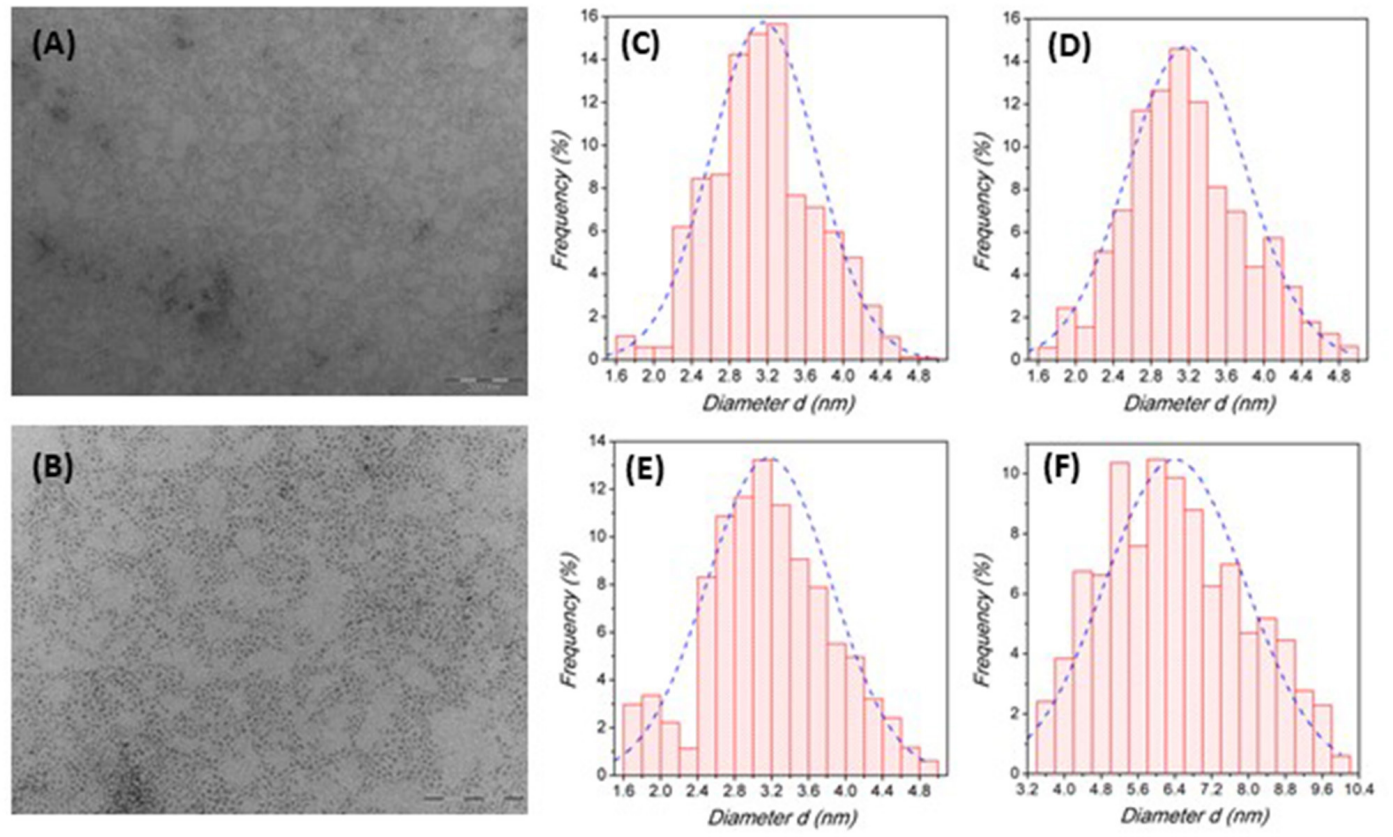
**(A,B)** TEM image of Cs_2_SnI_6_ nanocrystals at 100kx **(A)** and 250kx **(B)** magnifications. **(C–E)** Size distribution histograms for Cs_2_SnI_6_ **(C)**, Cs_2_SnBr_6_ **(D)**, Cs_2_Sn(I_0.5_Br_0.5_)_6_ **(E)**, and Cs_2_SnCl_6_ **(F)**, respectively, quenched after 1 min of reaction time.

The transmission electron microscopy (TEM) investigation performed on the samples revealed the presence of spherical NCs with an average particle diameter, *d*, of about 3.2 nm for all the compositions of the Cs_2_SnX_6_ system except for Cs_2_SnCl_6_ nanocrystals which showed a greater particle size of about 6.4 nm. Such nanocrystal size is similar to that reported by Wang et al. and Ghosh et al. for Cs_2_SnI_6_ and smaller than the recently reported data (Wang et al., 2016; Ghosh et al., 2018; Jing et al., 2019; Lin et al., 2019). Figures 2A,B report representative TEM images for the Cs_2_SnI_6_ crystals at two magnifications (other TEM images in the Supplementary Material), while Figures 2C–F report the size distribution diagrams for the four compositions reacted for 1 min.

Overall, the size distribution for Br- and I-based samples is quite narrow and symmetric as for the mixed I/Br sample, while for Cs_2_SnCl_6_ there is a clear evidence of a broader distribution. As reported in the Experimental Section, all these samples have been prepared by quenching the reaction after 1 min following the injection of the Cs-oleate precursor. We explored different quenching times (for the Cs_2_SnI_6_ sample) by stopping the reaction at 15 s, 1 min (data reported in Figure 2C), 2 and 10 min after precursor addition (size distribution diagrams and TEM images reported in the Supplementary Material). Again, the samples present a spherical shape with a size of about 2.9 nm for a reaction time of 15 s, and of about 3.2 nm for all the other times (data reported in the Supplementary Material). The results from TEM investigation on this series of samples (Figure S3) indicate that the formation of NCs is fast and occurs within few seconds after the precursor injection, while the crystal growth, on the contrary, is very slow and requires long times to produce significant variations in the particle diameter (no variation in the morphology—at least on the time-scale explored). The reduced growth rate can be attributed to the action of the ligands on the NC synthesis: it was observed, indeed, that using smaller amounts of capping agents resulted in spherical crystals with a diameter of 15–20 nm (Ghosh et al., 2018; Xu et al., 2019). This result is coherent with previous works on Cs_2_SnI_6_ NCs reporting that no NCs were obtained when oleic acid was the only capping agent used during the synthesis, thus suggesting a suppressing action of this ligand on the crystal growth (Wang et al., 2016). Dolzhnikov et al., instead, carried out their syntheses without using any organic ligand, and the samples prepared at 220°C were reported to have a diameter of about 38 nm (Dolzhnikov et al., 2017). A very recent work, on the other hand, made use of oleic acid only, without any amine, leading to Cs_2_SnI_6_ NCs with an average size of 10–15 nm (Xu et al., 2019). It should be also noted that a narrow size distribution has been observed in most samples and for any reaction time, with standard deviations of 0.6 nm, except for the sample where the reaction has been stopped after 15 s (e.s.d. 1.2 nm), where a significantly broader size distribution has been observed. For stoichiometric samples, Cs_2_SnCl_6_ shows, along with a bigger particle size, also a greater standard deviation with respect to I and Br containing NCs (Figure 2F). However, the general good focusing of particle size together with the slow crystal growth can be very useful to achieve a good control over the NC size.

**Figure 3:**
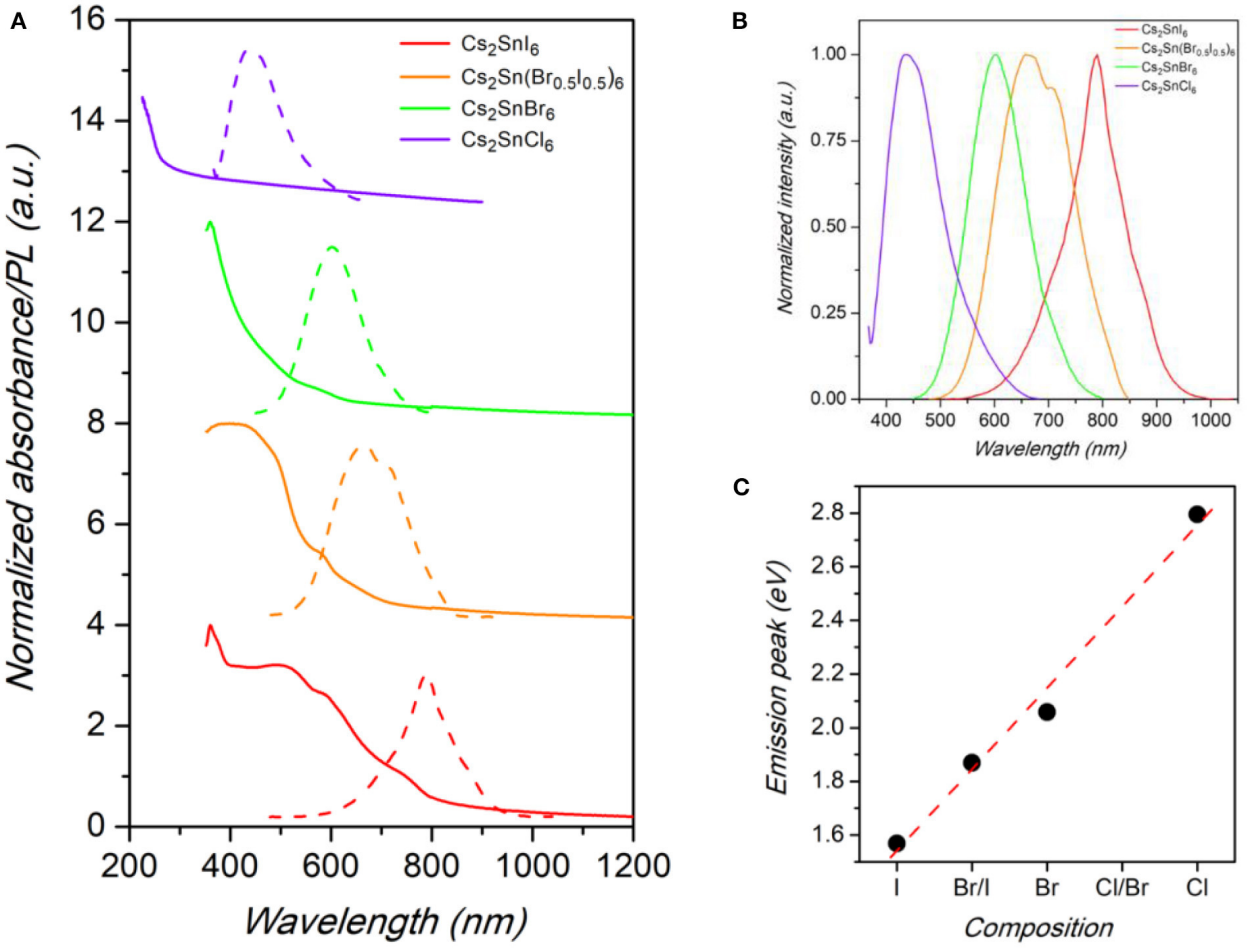
**(A)** Absorbance (solid lines) and PL spectra (dashed lines) of Cs_2_SnX_6_ (*X* = Cl, Br, Br_0.5_I_0.5_, and I) samples. **(B)** Comparison between the normalized PL spectra of Cs_2_SnX_6_ (*X* = Cl, Br, Br_0.5_I_0.5_, and I) samples. **(C)** Trend of emission peak energy vs. chemical composition in the samples. Dashed line is a guide for the eye.

The optical properties of Cs_2_SnX_6_ NCs were characterized by UV-vis-NIR absorbance and photoluminescence (PL) spectroscopy. Unlike lead halide NCs, that usually display a sharp absorption edge, the spectra were found to have a less distinct profile with a long tail pushing toward longer wavelengths (Figure 3A). In this regard, they are similar to the absorbance spectra of CsSnX_3_ NCs (Jellicoe et al., 2016). These results have been previously attributed to the presence of shallow electronic states, arising from crystal defects, such as halide vacancies, that have a low energy of formation and which are believed to introduce such states below the CB edge (Xiao et al., 2015; Maughan et al., 2016; Saparov et al., 2016).

The emission spectra of the Cs_2_SnX_6_ NCs (X = Cl, Br, Br_0.5_I_0.5_, I), reported in Figures 3A,B, are peaked at 444 nm (2.80 eV), 603 nm (2.06 eV), 663 nm (1.87 eV), and 790 nm (1.57 eV), by changing the halide content from Cl to I, with corresponding full-width at half maximum (FWHM) of 117, 122, 165, and 112 nm, respectively. The values we found for Cs_2_Sn(Br_0.5_I_0.5_)_6_ and Cs_2_SnI_6_ NCs are considerably higher than the fundamental gap energies reported for these compounds in the bulk form (1.43 and 1.3 eV, respectively), thus suggesting a significant blue-shift in the NC optical properties due to quantum confinement effects (Kaltzoglou et al., 2015, 2016; Yuan et al., 2019). The values of the band gap extracted from the Tauc plot of the absorbance spectra are reported in Table S1 of the Supplementary Material and confirms the scaling of the emission with a possible dependence of the Stoke shift with the halide nature. The trend of band gap of NCs compared to bulk samples is consistent with TEM investigation, that in fact revealed a very small diameter for the present crystals, and with previous studies on Cs_2_SnI_6_ NCs (Wang et al., 2016; Dolzhnikov et al., 2017; Xu et al., 2019). In the case of Cs_2_SnBr_6_ and Cs_2_SnCl_6_, instead, the PL peak energies are lower than the reported values of the bulk perovskites but in line with a linear scaling of the energy-gap variation with composition (see Figure 3C), thus suggesting a more significant impact of size reduction for these systems (Kaltzoglou et al., 2016). We highlight that the FWHM of the samples reported in this work, while larger with respect to state-of-the-art lead halide nanocrystals (typically 30–40 nm or even lower), are smaller than the values for the only published, to date, sample of the series investigated here, i.e., Cs_2_SnI_6_, being of the order of 150–200 nm (Wang et al., 2016; Dolzhnikov et al., 2017; Xu et al., 2019).

Estimation of the quantum yield (QY) has been carried out as reported in the Experimental section. For all the NCs synthesized and characterized in the present work, the QYs range around 0.4–1.4%. Such values are quite low if compared to lead analogs, but improved with respect to the value of 0.48% reported for Cs_2_SnI_6_ NCs by Wang et al. (2016).

**Figure 4:**
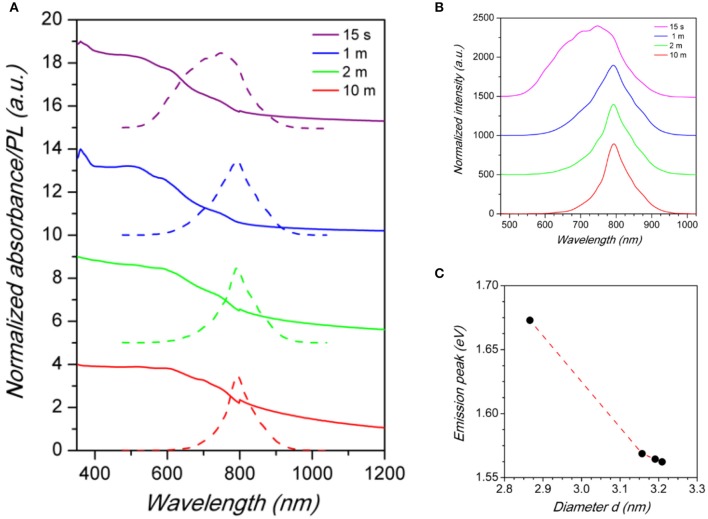
**(A)** Absorbance (solid lines) and PL (dashed lines) spectra of Cs_2_SnI_6_ samples as a function of reaction time after Cs-oleate addition (reported in the legend). **(B)** Comparison between the normalized PL spectra of Cs_2_SnI_6_ for different reaction times. **(C)** Emission peak energy vs. Cs_2_SnI_6_ nanocrystal size. Dashed line is a guide for the eye.

Absorption and emission optical measurements have been as well carried out on the Cs_2_SnI_6_ series as a function of NCs reaction time discussed above (Figure 4A).

**Figure 5:**
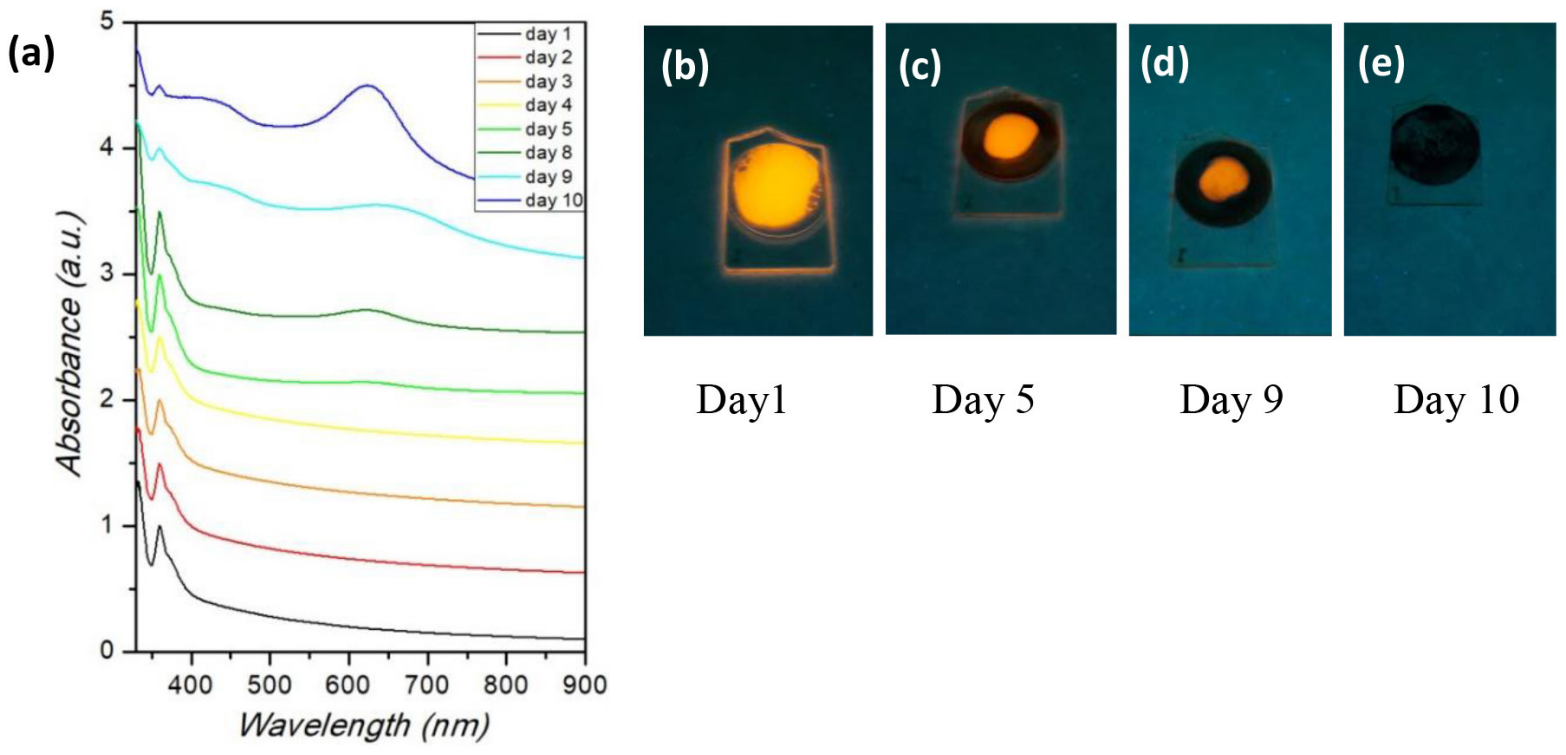
**(a)** Absorbance spectra of Cs_2_SnI_6_ samples as a function of air-exposure time; **(b–e)** Photos of Cs_2_SnI_6_ samples under UV-lamp as a function of air-exposure time.

The PL spectra reported in Figure 4B reveal that the size of NCs has an impact on the luminescence properties of Cs_2_SnI_6_. By increasing the particle diameter from about 2.9 nm to about 3.2 (determined by TEM, see Supplementary Material), a red-shift of the emission peak is observed from about 741 nm (1.67 eV) to 793 nm (1.56 eV) (Figure 4C) which is a huge variation of the optical properties for a relatively small variation of the nanocrystal size. Moreover, crystal size affects as well the FWHM of PL spectra: the shift of the peak wavelength is indeed accompanied by a narrowing of the emission linewidth. As a matter of fact, the sample with the smallest NCs size presented the broadest peak of the series, with a FWHM of ~200 nm, while, on the other hand, the sample reacted for 10 min has the narrowest one, with a FWHM of 79 nm (see the size distribution plots in the Supplementary Material). These results, due to quantum confinement effects arising from the small particle diameter, demonstrated that the luminescence properties of the Cs_2_SnX_6_ NCs can be tuned, with the present synthetic protocol, by varying both their chemical composition and crystal size. Finally, and concerning air-stability, we highlight that the present nanocrystals, deposited on a glass slide exposed to ambient conditions, retained their emission properties for about 10 days, thus already suggesting a good starting point to further enhance their stability by playing with the synthetic routes. This can be appreciated in Figures 5a–e reporting the absorbance as a function of time up to 10 days, as well as some selected photos at four time intervals (Day 1, 5, 9, and 10). Absorbance and UV-lamp photos (excitation at 365 nm) clearly show that up to 9 days of air exposure the spectra are unchanged and the red-emission is still evident. On the other hand, the spectra at Day 10 shows the appearance of a new profile peaked at about 600 nm deriving from decomposition products which is slightly observable already at Day 8, suggesting partial decomposition from this time.

## Conclusions

Nanocrystalline Cs_2_SnX_6_ (X = Cl, Br, Br_0.5_I_0.5_, and I) samples have been prepared by a simple, optimized, hot-injection method without the use of toxic phosphines, reporting for the first time the synthesis of Cs_2_SnBr_6_, and mixed Cs_2_Sn(I_0.5_Br_0.5_)_6_ samples. The synthetic approach allowed to finely tune the anion composition and to prepare single-phase cubic samples with a linear scaling of the unit cell volume along with the I/Br content. The prepared nanocrystals, for the same reaction time, have a spherical shape with average size in the range around 3–6 nm, depending on the composition. Optical properties have been nicely modulated by the halide content by progressively tuning the optical emission from ~440 nm for Cs_2_SnCl_6_ to ~790 nm for Cs_2_SnI_6_, also showing that Sn^4+^-based perovskites-derivatives have a lower energy gap, when compared to their lead-based counterpart, reaching the NIR region; this contributes to make them potentially better materials for photovoltaic applications and widen the optical range of this class of known materials. In addition, the prepared samples showed stable emission properties under ambient conditions for several days. By varying the reaction time from 15 s to 10 min the nanocrystal size showed only a limited growth (from about 2.9–3.2 nm) which however resulted in a progressive red-shift of about 0.1 eV of the band gap as well as in a reduction of the PL spectra FWHM from 210 to about 80 nm. The present results add a significant contribution toward the discovery of new lead-free nanocrystalline phases with enhanced air-stability by showing, for the first time, an easy and reliable tuning of optical properties of perovskite-derivatives by anion composition in analogy with the results available for 3D lead halide perovskites.

## Experimental Section

### Chemicals

Cs_2_CO_3_ (Acros Organics, 99.995%), SnCl_4_ (Sigma-Aldrich, 99.995%), SnBr_4_ (Sigma-Aldrich, 99%), SnI_4_ (Sigma-Aldrich, 99.999%), 1-octadecene (ODE, Acros Organics, 90%), oleic acid (OA, VWR Chemicals, 81%), oleylamine (OLA, Acros Organics, 80–90%), hexane (HEX, Sigma-Aldrich ≥99%), 1-butanol (BUT, Sigma-Aldrich, ≥99.4%). All reagents were used without further purification.

### Synthesis

#### Synthesis of Cs-Oleate

Fifteen milliliters of ODE was loaded in a 50 mL three-neck flask along with 0.5 mL of OA and 0.5 mL of OLA and dried under vacuum for 1 h at 120°C. After that, 0.1625 g of Cs_2_CO_3_ was added to the reaction vessel and the mixture was dried again under vacuum for 1 h at the same temperature. Finally, the flask was heated at 150°C under nitrogen flow until complete dissolution of Cs_2_CO_3_. The resulting solution was stored under nitrogen atmosphere. Since Cs-oleate is insoluble in ODE at room temperature, we preheated the solution at 130°C to dissolve the precipitate before use.

#### Synthesis of Cs_2_SnX_6_ NCs

ODE (5 mL), OA (0.2 mL), and OLA (0.2 mL) were combined in a 50 mL three-neck flask and dried under vacuum for 1 h at 120°C. Subsequently, 0.235 mmol of SnX_4_ (0.1468 g of SnI_4_, 0.1027 g of SnBr_4_, and 0.0611 of SnCl_4_, respectively) was added to the flask and the solution was degassed under vacuum for 15 min at 80°C. In the case of mixed-composition NCs, an equimolar ratio of SnBr_4_ and SnI_4_ was used. The flask was then heated at 220°C under nitrogen flow and 1.5 mL of the as-prepared Cs-oleate solution was quickly injected with vigorous stirring. After a specific reaction time (15 s, 1 min, 2 min, 10 min), the mixture was cooled down in an ice bath. To purify the NCs, the mother solution was centrifuged at 7,000 rpm for 5 min and the surfactant was discarded. Some of the crystals were deposited on glass slides and covered with cover-slips, while the remaining crystals were dispersed in 5 mL of HEX. No argon-filled glovebox was needed during the synthesis and the purification process, nor to store the final samples. The stoichiometry of the samples was checked with Energy Dispersive X-ray (EDX) spectroscopy and was found to be in agreement with the nominal one within an e.s.d. of 5%.

### TEM

Transmission electron microscopy (TEM) was performed on a JEOL JEM-1200 EX II microscope operating at 100 kV, equipped with a tungsten filament as electron source. The specimens were prepared by depositing 8 μL of NCs in HEX on an aluminum grid covered with a polymeric film and letting the solvent evaporate under ambient condition. The size distribution of the samples was calculated from TEM images using ImageJ software.

### XRD

X-ray diffraction (XRD) investigations were carried out under ambient condition on a Bruker D8 Advance diffractometer using copper Kα radiation (λ = 1.54056 Å) as X-ray source. The measurements were performed in the Bragg-Brentano configuration, with resolution of 0.04° and integration time of 4 sec. The specimens were prepared by depositing the NCs on a glass slide and covering them with a glass cover slip. The reference patterns and crystal constants were calculated using PowderCell software. Lattice parameters were determined from Rietveld refinement of the diffraction pattern.

### Absorption Spectroscopy

Ultraviolet-Visible-Near Infrared (UV-Vis-NIR) absorption measurements were performed under ambient conditions using a Varian Cary 6000i spectrophotometer equipped with a double monochromator, a deuterium lamp and a tungsten filament lamp as light sources, a photomultiplier (UV-Vis) and an InGaAs photodiode (NIR) as detectors. Spectral range was 200–1,800 nm, in step of 2 nm. The specimens were prepared by depositing the NCs on a glass slide and covering them with a glass cover slip. The baseline was taken using the glass slide as reference.

### Steady-State PL Spectroscopy

PL investigations were conducted under ambient conditions using a 355 nm laser line (PowerChip Microlaser) as excitation light source and a LN2-cooled silicon CCD (Princeton Instruments Spec-10:400) as detector. The specimens were prepared by depositing the NCs on a glass slide and covering them with a glass cover slip. Measurements were performed in transmission mode, with a resolution of 0.5 nm and an integration time of 5 sec, using a longpass filter at 450 nm to remove the laser line from the PL signal. The background noise and the luminescence from the glass slide were subtracted from the spectra of the samples. Since PL signals were not perfectly symmetric Gaussian curves, the emission peak positions were determined by fitting the spectra a Gaussian lineshape.

### Photoluminescence Quantum Yield

PL QY measurements were performed on the samples dispersed in HEX into quartz cuvettes (1 cm optical path) using a Varian Cary Eclipse Fluorescence Spectrophotometer with an excitation wavelength of 350 nm. We used Rhodamine B and Rhodamine 6G in ethanol as standards to calculate the QY values for the NCs.

### Energy Dispersive X-Ray Spectroscopy

Energy dispersive x-ray spectroscopy (EDX) has been performed with a high-resolution scanning electron microscope (SEM, TES- CAN Mira 3) operated at 15 kV. For all the samples it was found an experimental cation/anion ratio in agreement (within a 5% error) with the nominal compositions. Also for mixed Br/I compositions the anion ration expected by the stoichiometry was confirmed experimentally.

## Data Availability Statement

All datasets generated for this study are included in the article/Supplementary Material.

## Author Contributions

AV and CC carried out synthesis and PL measurements. MP supervised the optical measurements and coordinated the work. PQ supervised the material synthesis. DB contributed to spectroscopic measurements. LM devised the work and coordinated the whole activity. LM and MP wrote the manuscript.

### Conflict of Interest

The authors declare that the research was conducted in the absence of any commercial or financial relationships that could be construed as a potential conflict of interest.
