# Supplementary material for: Highly Tunable Emission by Halide Engineering in Lead-Free Perovskite-Derivative Nanocrystals: The Cs2SnX6 (X = Cl, Br, Br/I, I) System
Source: Front Chem. 2020 Jan 31;8:35. doi: 10.3389/fchem.2020.00035 (PMC7004971; doi:10.3389/fchem.2020.00035)
Supplement: Supplementary file 1 [file Table_1.DOCX]

**SUPPLEMENTARY MATERIAL**

Highly Tunable Emission by Halide Engineering in Lead-free Perovskite-derivative Nanocrystals: the Cs_2_SnX_6_ (X=Cl, Br, Br/I, I) System

Alessandro Veronese,^a^ Maddalena Patrini,^a,*^ Daniele Bajoni,^b^ Carlo Ciarrocchi,^c^ Paolo Quadrelli,^c^ Lorenzo Malavasi^c,*^

^a^Department of Physics and CNISM, Università degli Studi di Pavia, Via Bassi 6, 27100 Pavia, Italy; ^b^Dipartimento di Ingegneria Industriale e dell’Informazione, Università degli Studi di Pavia, via Ferrata 1, 27100 Pavia, Italy; ^c^Department of Chemistry and INSTM, Università degli Studi di Pavia, Viale Taramelli 16, 27100 Pavia, Italy.


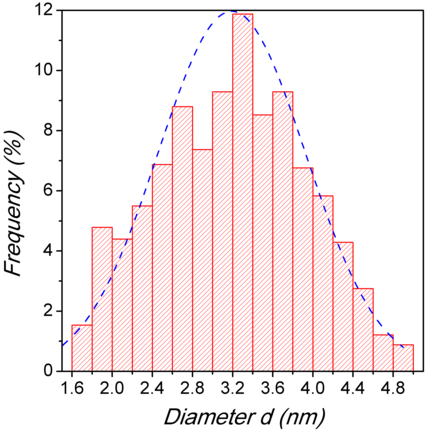

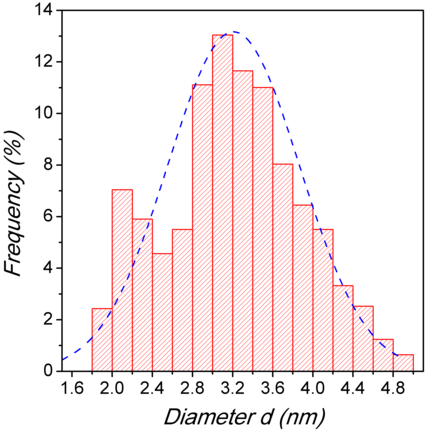


**Figure 1S**: Size distribution histograms for Cs_2_SnI_6_ quenched after 2 min (left panel) and 10 min (right panel) of reaction time.





**A**





**B**

**

**

**C**

**Figure 2S**: A) TEM image of Cs_2_SnBr_6_ nanocrystals; B) TEM image of Cs_2_Sn(Br_0.5_I_0.5_)_6_ nanocrystals, and C) TEM image of Cs_2_SnCl_6_ nanocrystals.





**A**

**B**








**C**

**Figure 3S**: A) TEM images of Cs_2_SnI_6_ nanocrystals reacted for 15 s (A), 120 s (B) and 600 s (C).

**Table 1**: Size distribution and QYs for the various samples reported in the work as a function of the halide and of reaction time.

| **Sample** | **X** | **Average Size (nm)** | **QY (%)** | **Band gap (eV)** |
| --- | --- | --- | --- | --- |
| Cs_2_SnX_6_ | I | 3.16 ± 0.56 | 1.36 | 1.57 |
|  | Br0.5/I0.5 | 3.18 ± 0.67 | 0.37 | 2.02 |
|  | Br | 3.18 ± 0.63 | 0.83 | 3.33 |
|  | Cl | 6.40 ± 1.53 | - | 3.86 |
|  |  |  | - |  |
| **Cs_2_SnI_6_** | **Reaction Time (s)** |  | - |  |
|  | 15 | 2.86 ± 0.65 | - |  |
|  | 60 | 3.16 ± 0.56 |  |  |
|  | 120 | 3.19 ± 0.73 |  |  |
|  | 600 | 3.21 ± 0.67 |  |  |
